# Report of the Scientific Committee Appointed to Investigate the Physiological and Therapeutical Effects of the Hypodermic Method of Injection

**Published:** 1867-12

**Authors:** 


### Miscellaneous.

### Report of the Scientific Committee appointed to Investigate the Physiological and Therapeutical Effects of the Hypodermic Method of Injection.

Without objecting to the word hypodermic, the Committee resolved to employ the term subcutaneous in their report. The conclusions have been drawn from experiments on animals and on man in health and disease; from personal evidence of experienced medical men given before the Committee; from records of facts and other communications in answer to a series of questions drawn up by the Committee. The subject intrusted to the Committee was the physiological and therapeutical effects of this method.

I.—*Physiological Division.* The first experiments were made to determine the quantity of water that can be injected under the skin. It was found that the quantity varies directly as the yielding and elastic quality of the skin at the locality injected. Watery solutions of drugs were used for injection, and it was found that neutral solutions, as a rule, were tolerated, but that very acid and very alkaline solutions were apt to cause irritation. Experiments were made for the purpose of comparing absorption by skin and vein, and it was found that a drug injected subcutaneously was far less rapidly absorbed and less intense in its effects than when it was introduced into a vein. In the numerous experiments made by the Committee no symptoms have arisen which would lead them to conclude that the drug subcutaneously injected had been thrown into a vein. The pain of injection was found to depend to some extent on the density of the skin; the less the resistance presented to the needle, the less the pain presented on the puncture. The Committee directed their attention to the effects of this method of administering various drugs, as compared with those methods in general use, viz: the mouth and the rectum—and the special points examined were, the relative rapidity of absorption, the intensity and duration of the effects following each method of administration. The following alkaloids were used: Aconitia, atropia, morphia, strychnia, and quinia. Experiments were also made with Calabar bean, conia, hydrocyanic acid, iodide of potassium, podophyllin, colocynth, aloes, and Battley's solution of opium. In the experiments with aconitia on animals the local action of the drug was exhibited in different ways, though the general type of symptoms was the same by the three methods—by the mouth the drug affected the salivary glands, by the rectum it irritated the gut, by the skin it gave rise to local pain. The smallest dose found to produce death in rabbits was—by the mouth, $\frac{1}{60}$th gr.; by the rectum, $\frac{1}{50}$th gr.; by the skin, $\frac{1}{300}$th gr. With atropia it was found that there was a stage of excitement which followed the subcutaneous injection of the drug, and was a remarkable feature of this method. Tables of the effects of this drug on man were given, and it was found that $\frac{1}{100}$th of a grain subcutaneously was sufficient to accelerate the pulse considerably. The comparative effects of morphia by the three methods were then described; the train of symptoms was found to be closely similar. A table of the effects on rabbits and on man was given in a concise form, showing that the effects of the drug injected under the skin were more rapidly manifested and more intense than by the other methods. Some interesting results were obtained from the experiments made with quinia on man. When the drug was injected into the cellular tissue, considerable elevation of the temperature was observed, this symptom being slight or inappreciable when the drug was taken (in the same quantity) by the mouth or by the rectum. Series of experiments were made in a similar manner with Calabar bean, coniastrychnia, and hydrocyanic acid, and the results obtained were tabulated in a convenient form. Experiments were also made with iodide of potassium on a healthy man who had congenital extroversion of the bladder; the drug produced some local irritation, which prevented the completion of the series. A solution of podophyllin injected under the skin was found to give rise to free diuresis—a symptom which was characteristic of this method of administering the drug. Experiments were also made with solutions of colocynth and aloes, but considerable irritation followed their use.

II.—*Therapeutical Division.* The Committee were limited in the number of drugs that could be used, from the locally irritating properties which some valuable medicines possess. Although many experiments were performed to test the value of local injections, the Committee failed to obtain any evidence to show that the local predominates over the general effects. Investigations were then made of the therapeutical value of this method of administering various drugs. Aconitia was found to give rise to so much local tingling that the drug was considered unfit for subcutaueous injection. In case of simple neuralgia, atropia was considered to have a very beneficial effect when thus given, and in some cases more permanent relief was found to follow its injection than that of morphia. The Committee believed that the value of morphia was materially enhanced by this method, as the action of the drug is not only secured with greater intensity and rapidity than by the ordinary modes, but the duration of its effects is prolonged. The same advantages characterize this mode of giving quinia in intermittent fevers, but some caution is requisite in giving large doses of this drug, as irritation may arise from its presence under the skin. The conclusions which the Committee deduce from their investigations were—1. That, as a general rule, only clear neutral solutions of drugs should be injected, for such solutions rarely produce local irritation. 2. That, whether drugs be injected under the skin, or administered by the mouth or rectum, their chief physiological and therapeutical effects are the same in kind, though varying in degree. 3. But that symptoms are observed to follow the subcutaneous injection of some drugs which are absent when they are administered by the other methods, and, on the other hand, certain unpleasant symptoms which are apt to follow the introduction of the drugs by the mouth and rectum are not usually experienced when such drugs are injected under the skin. 4. That, as a general rule, to which, however, there may be exceptions, clear neutral solutions of drugs introduced subcutaneously are more rapidly absorbed and more intense in their effects than when introduced by the rectum or the mouth. 5. That no difference has been observed in the effects of a drug subcutaneously injected, whether it be near to, or at a distance from, the part affected. 6. That the advantages to be derived from this method of introducing drugs are—*(a)* rapidity of action; *(b)* intensity of effects; *(c)* economy of material; *(d)* certainty of action; *(e)* facility of introduction in certain cases; *(f)* with some drugs the avoidance of unpleasant symptoms. This plan, therefore, is most likely to be adopted where very rapid and decided effects are required from drugs which are operative in small doses.—*Medical Times and Gazette.*

